# Covering, corner-searching and occupying: A three-stage intelligent algorithm for the 2d multishape part packing problem

**DOI:** 10.1371/journal.pone.0268514

**Published:** 2022-05-31

**Authors:** He Ren, Rui Zhong

**Affiliations:** School of Astronautics, Beihang University, Beijing, China; National Institute of Technology Silchar, India, INDIA

## Abstract

The bin packing problem has a wide range of applications in industry. With the upgrade of the task difficulty, the traditional 2d rectangular layout algorithm can no longer meet the needs of modern industry, such as express packing task and exoplanet ore collection task. The express or ore samples come in heterogeneous shapes so they cannot all be treated as rectangular pieces. In this paper, we propose a three-stage method called covering, corner-searching and occupying (C,S&O) to solve the two-dimensional multishape part packing problem. The objective of the packing problem variant is to ensure maximum use of the raw material and minimize the trim loss. The algorithm cannot make use of information about the sequence of future objects that are going to arrive, only knowing the shape and size of the coming one, and the coming part must be packed into the bin immediately after its arrival without buffering or readjusting. The method of C,S&O hybridizes the idea of “gold corners, silver edges and grass belly” in the Chinese game Go and the method of finding picture corners in machine vision. In the first stage, the rectangular bin and the coming part are transformed into matrix representation, and generating the position matrix that indicates possible ways of packing the part into the bin. In the second stage, the suitable layout position of the coming part is obtained using machine vision image processing technology for reference. The third stage is calculating the environment matching degree to determine the current optimal placement orientation. In order to facilitate the display of the simulation results, only three shapes of parts are considered in the simulation, rectangle, circle and triangle. The experimental results show the effectiveness of this method. Consulting the literature, it is found that this paper is the first to propose a layout method for multishape manufacturing parts.

## Introduction

The two-dimensional bin packing problem is an important combinatorial optimization problem that has been widely worked by many researchers. The investigation of this problem has an important implication for many industries [1, 2], such as textiles, paper, glass, metal, leather, transportation, etc. An excellent algorithm can provide increasing of the profitability by reducing raw material waste and accelerating production efficiency. However, even though this issue is decades old, many layout tasks are still executed by workers on the basis of their own experience and judgment [3]. The actual engineering environment has many uncertainties compared to the computer simulation environment, such as breakages in rectangular raw materials, the movement of the raw materials due to the vibration of the working platform, and changes in the production task requirements, resulting in the need to spend much time modifying the program. The manual layout process often fails to yield a satisfactory layout solution, which will make the waste of a large amount of valuable raw material. On the other hand, many modern packing tasks, such as express transportation, exoplanet ore sampling, rocket intelligence assembly, etc., put forward higher requirement for layout technology. The parts to be arranged are of different shapes and cannot be treated simply as rectangular or circular parts. The currently proposed layout algorithms have difficulty dealing with this type of layout problem.

Bin packing problems not only have important applications in industry field but are also at the heart of many interesting fields. Guasque and Ana aim to use bin packing algorithms for partition allocation to save energy [4]. Danqing Feng, Zhibo Wu et al. mention that researchers have solved virtual machine placement problems by using bin packing algorithms to meet users’ flexible demands in cloud computing [5]. For a bioinformatic problem in which the sequencing alignment process is computationally challenging, Mohamadi, Hamid et al. propose a distributed and parallel indexing and alignment framework to solve the bin packing problem [6].

The objective of the 2d multishape part packing problem is to maximize the utilization of raw materials, requiring that the parts in the arrangement cannot overlap with or cross each other. The problem is strongly NP-hard since it generalizes the one-dimensional bin packing problem [7, 8]. Many researchers have done a great deal of work on the bin packing problem [9]. The existing methods can be boardly classified into categories, online and offline. The online methods do not have the knowledge of all objects to be packed and hence, are comparatively difficult to solve. Nestor M. Cid-Garcia and Yasmin A. Rios-Solis propose an exact approach called positions and covering to solve the two-dimensional bin packing problem. The algorithm can obtain good solution for the rectangular packing problem considering the rotation cases [10]. And they also use a similar way to address the 2D strip packing problem [11]. The bottom-left heuristic algorithm was proposed by Baker, Coffman and Rivest [12], which places the part in the bottom-left conner of the bin and moves it to get the solution, and the computation burden is very heavy when there are a lot of parts in the bin. A. K. Sato, T. C. Martins et al. use an obstruction map to search for the optimal solution for the 2D irregular bin packing problem [13], which reduces the search space by enforcing a grid placement restriction for the items. Sergey Polyakovskiy and Rym M’Hallah aim to model the difficult discrete optimization problem as an integrated constraint program augmented with two sets of logically redundant constraints that speed up the search [14]. Christian Blum, Verena Schmid et al. hybridize a randomized multi-start version of a constructive one-pass heuristic with an evolutionary algorithm to address the oriented two-dimensional bin packing problem under free guillotine cutting [15]. The algorithm is hart to deal with large size parts. Yaodong Cui, Yi Yao et al. present a two-phase heuristic approach for a bin packing problem with two-stage patterns and nonoriented items, which generates a solution in each phase which makes the algorithm time-consuming [16]. A lot of researchers like to use meta-heuristic algorithm to get a global optimal solution, such as the genetic algorithm [17], simulated annealing [18], and tabu search [19]. These methods are typical offline methods, which assume that the information of all objects is known. The objective of this kind of algorithm is to get a global optimal solution, so the meta-heuristic algorithm needs to spend a lot of time to calculate. Nowadays artificial intelligence methods have performed well in many fields. In particular, reinforcement learning achieves human level decision-making on some tasks. This has encouraged a few researchers to apply deep reinforcement learning (DRL) to solve bin packing problems. Olyvia Kunku, Samrat Dutta et al. propose a deep reinforcement learning framework based on a double deep Q-network (DQN) to solve the online bin packing problem that takes an image showing the current state of the bin as input and gives out the pixel location of the part [20]. The algorithm can process the incoming part immediately, but the sample types that can be processed are single. Alexandros Bouganis and Murray Shanahan present an automated system that utilizes computer vision and artificial intelligence techniques to solve packing problems without human interference [21]. The algorithm doesn’t consider the rotation case, and if there is extend interference in the working process the algorithm cannot output the result.

Although the bin packing problem has certain applications in actual production, with the emergence of intelligent, high-capacity joint production lines in modern industrial production, a variant of the rectangular part layout problem, the multishape manufacturing part layout problem, is gaining increasing attention and has not been explored. This problem accords better with the needs of modern production. To date, few scholars have studied this problem, which is more complex than the rectangular part layout problem. In view of the above, this paper proposes an intelligent algorithm to solve the layout problem for rectangular, right triangular and circular parts. There are three main contributions in this paper. Firstly, a novel overlapping detection method is proposed which is more simpler and more convenient than no-fit polygon [22]. Secondly, a new scoring rule has been proposed to choose the best-fit location of the part to be placed. Referring to the way of neural network processing picture, the correct position of the parts to be placed can be obtained in real time. Thirdly, with the help of image processing technology, parts or raw material bin of arbitrary shape can be transformed into matrix representation. So this algorithm can deal with the optimization problem of packing parts of arbitrary shape on raw material of arbitrary shape, and it is more robust than existing algorithm.

The rest of this paper is arranged as follows: In section *Problem description and materials*, we present the mathematical model used in this paper. *In section The method, the details of the method will be introduced. In section Experiment, we give out* the experimental simulation results, Finally, an outlook to the future and conclusions are given in section *Conclusion*.

## Problem description and materials

**Fig 1:**
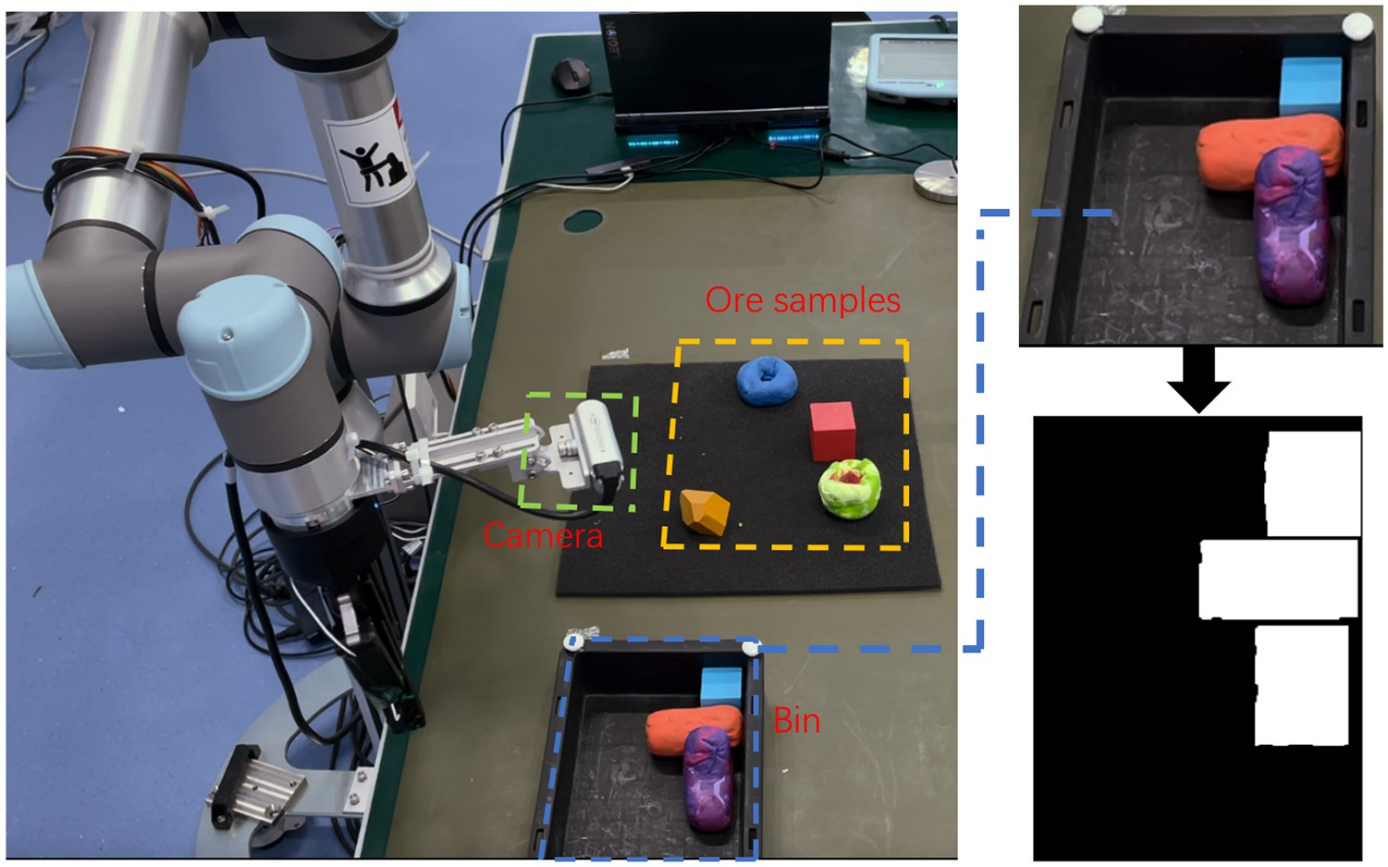
Experiment. In order to verify the effectiveness of the algorithm, an physical experiment is carried out in this paper. Use a robotic arm to collect samples and place them into a bin. The top view of the bin is obtained by hand-eye camera, and then the top view is converted into a binary image.

We consider a real production scenario where the robot needs to collect ore samples and place them properly into the bin. The agent can only know the shape and size information of the sample it grabs. And the agent can use the hand-eye camera to obtain the occupancy of the bin before each sample placement. The job of the agent is to quickly and accurately determine the location and orientation of the parts to be packed in the rectangular bin, at the same time to maximize the bin occupancy rate. The larger the bin occupation, the higher the work efficiency, the great the overall benefit. In this article, we use rectangles, triangles and circles to represent the shapes of different ores, because most ores can be classified into these three cases. The simulation experiment is shown in Fig 1.

**Fig 2:**
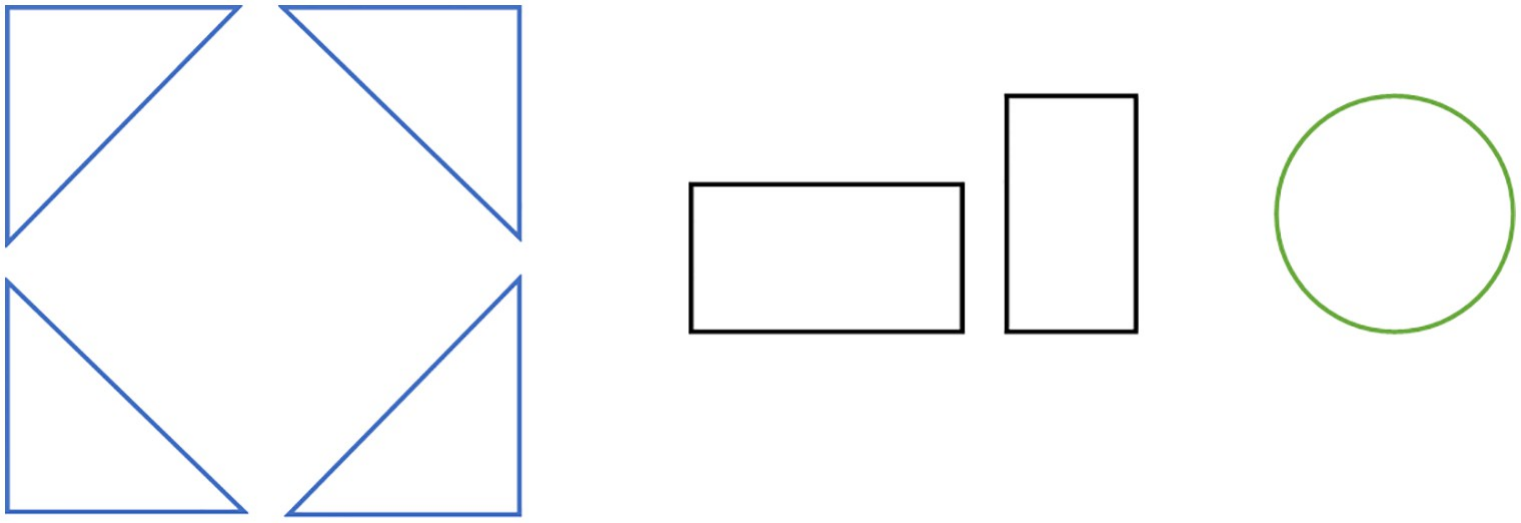
Rotation rules. In this paper, triangular parts are set to have four layout orientations, rectangular parts have two layout orientations, and circular parts have one layout orientation.

Therefore, the problem is described concretely as follows: the dimensions of the rectangular bin are fixed, with length *L* and width *W*. There is a set of two-dimensional parts that need to be packed in the rectangular bin, which will be represented by a set ***I***. In theory, the size of ***I*** is infinite. Each part *p*_*i*_ ∈ ***I*** has specific shape and size information, $p_{i}=[\begin{array}{ccc} c_{i} & l_{1} & l_{2} \end{array}]$, where *c*_*i*_ represents the shape of the part. This paper specifies 0 for circles, 1 for rectangles, and 2 for triangles, *l*_*i*_ and *l*_2_ represent the dimension information of the part. For a rectangular part, *l*_1_ = *h*, *l*_2_ = *w*; for a circular part, *l*_1_ = *l*_2_ = *r*; for a right triangular part, *l*_1_ and *l*_2_ are equal to the side lengths of the two right-angled sides. Additionally, when laying out the sample, rectangular parts have two layout orientations, triangular parts have four layout orientations, and circular parts have one orientation, as shown in Fig 2.

The ultimate goal of the task is to maximize the utilization *η* of the rectangular bin. Let $I=\{\begin{array}{ccc} p_{1} & \cdots & p_{n} \end{array}\}$ be the set of parts packed into the rectangular bin; then, we have:

$$\begin{array}{c} \text{max}(\eta )=(\frac{\sum_{1}^{n}a_{i}}{H\times W}\times 100\%) \end{array}$$ (1)

where *a*_*i*_ is the area of the part *p*_*i*_ in Eq (1).

### Matrix representation

**Fig 3:**
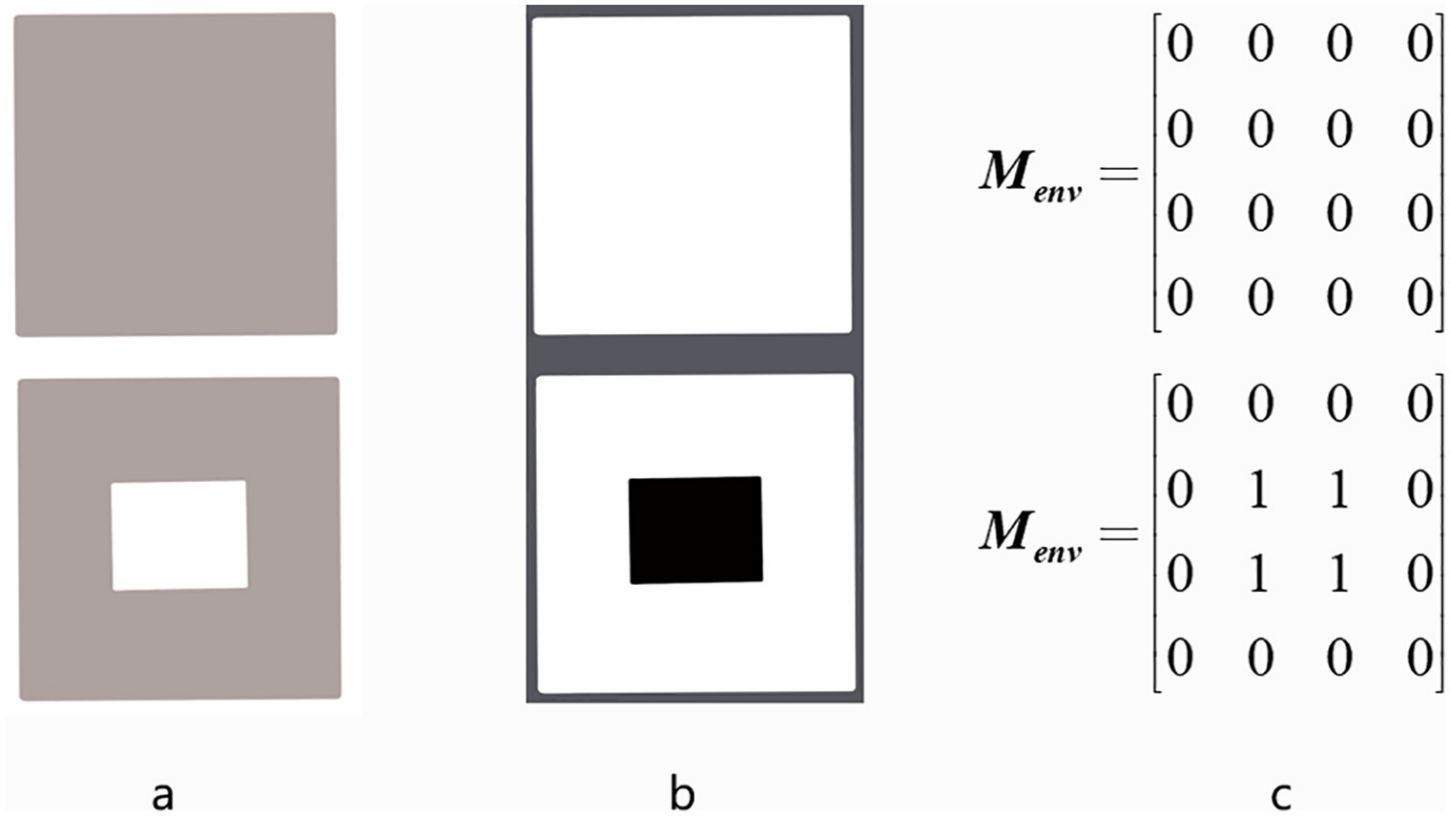
The way to get environment matrix. Image processing techniques are used to transform the top view of the bin into an environment matrix. The size of the environment matrix is the same as the pixel size of the photo.

To reduce the search space, an environmental grid model and a part matrix representation are proposed in this paper. For the environment grid model, we parameterize the bin through discretizing its bottom area as a *L* × *W* regular grid along length(*X*) and width(*Y*) directions, respectively. And we record at each grid cell the current utilization of the bin space, leading to an environment matrix ***M***_*env*_. In the matrix, element 1 indicates that the space of the bin is used or destroyed and 0 indicates the space of the bin is empty. The generation of the ***M***_*env*_ can be performed with the help of image processing techniques as shown in Fig 3.

**Fig 4:**
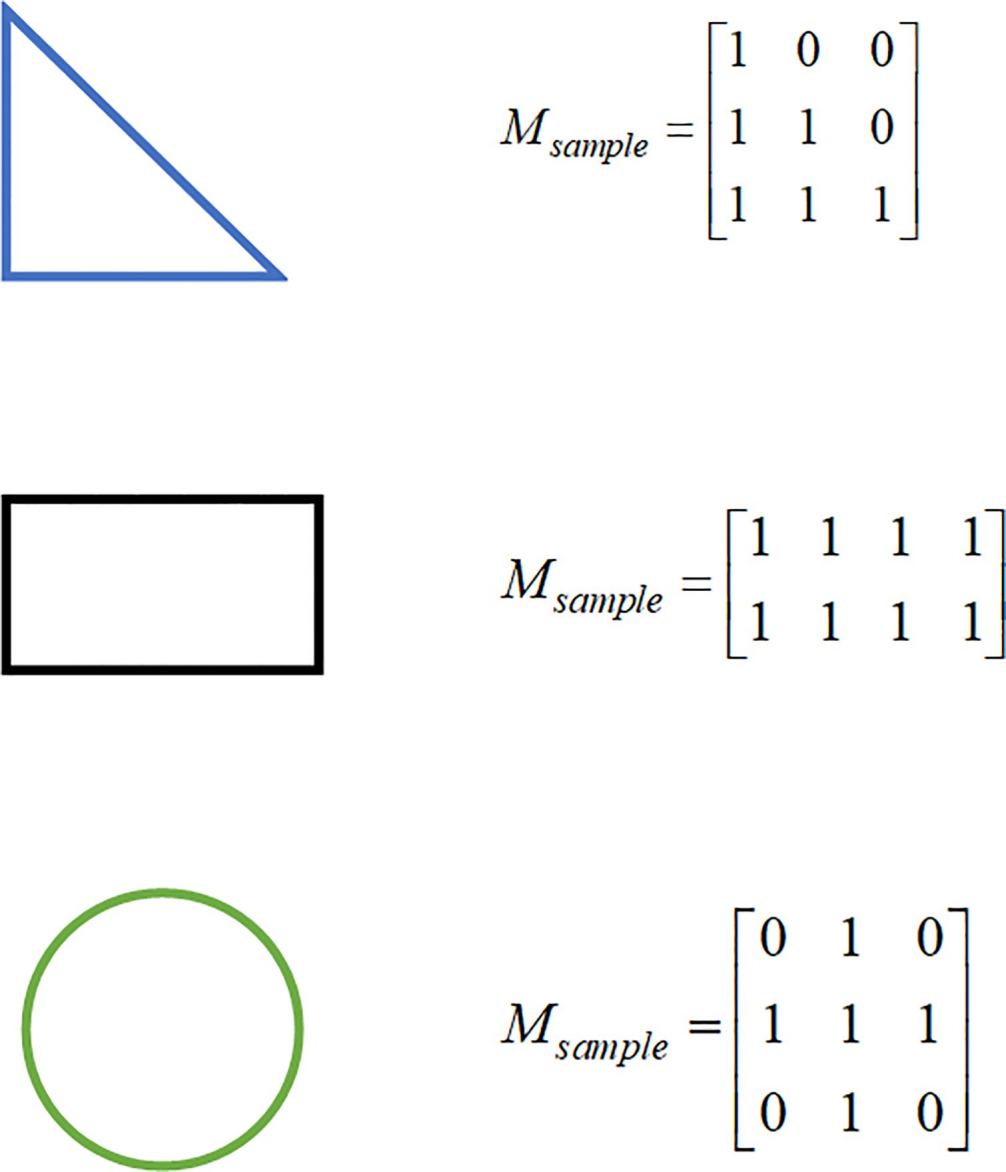
The sample matrix. Parts are represented as a sample matrix according to the part size information. The sample matrix size is the same as the smallest rectangle that can accommodate the part size.

Similarly, to improve the computational efficiency and facilitate overlap detection, this paper employs a part matrix representation to convert a part into a matrix, and we call the part matrix the sample matrix ***M***_*sample*_. The method is as follows: the part is placed in the smallest rectangle that can accommodate its size. Similar to the representation of the environment grid model, we parameterize the smallest rectangle through its surface as a *l* × *w* regular grid along the length (*l*) and width (*w*) dimensions. At each grid cell, we record a 1 or 0, where 1 means the grid cell contains the part and 0 means it is empty. The example is shown in Fig 4

### A novel overlapping detection method

**Fig 5:**
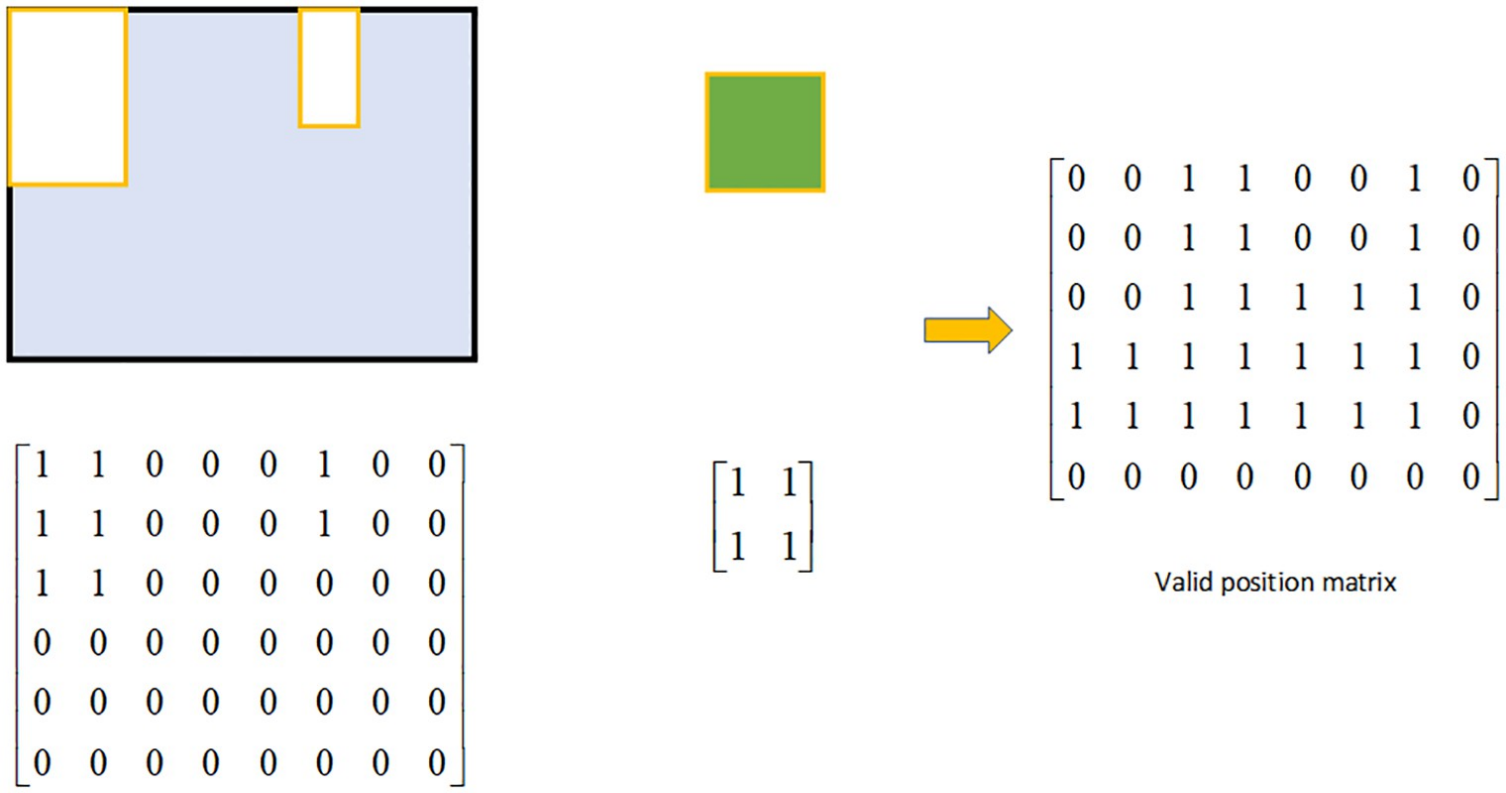
The way to get the valid position matrix. The bin and the parts to be placed are transformed into matrix representation, and the convolution operation is carried out by using the convolution neural network method, and finally the effective position matrix is obtained.

With the introduction of the environment grid model and the part matrix representation, it is simple to detect whether the part to be laid out overlaps with the already laid out parts. Inspired by convolutional neural network, a new overlapping detection method is proposed in this paper. In this method, the environment matrix ***M***_*env*_ is taken as the photo and the sample matrix ***M***_*sample*_ is taken as the convolution kernel. The convolution operation is performed on each position in the bin to obtain the valid position matrix ***M***_*vp*_. Element 0 in ***M***_*vp*_ represents the convolution result of this position is not 0, and the part placed at this position will overlap. Element 1 means this position in the bin will not overlap. The process is shown in the Fig 5

The calculation method of ***M***_*vp*_ is shown in Eq (2), where ◦ is the convolution operation, and *l* and *w* are the dimensions of the part to be packed.

$$\begin{array}{c} M_{vp}(i,j)=\{\begin{array}{c} \begin{array}{cc} 1 & ifM_{sample}◦M_{env}[i:i+l,j:j+w]=0 \end{array}\\ \begin{array}{cc} 0 & ifM_{sample}◦M_{env}[i:i+l,j:j+w]\neq 0 \end{array} \end{array} \end{array}$$ (2)

### The packing rule

In this paper, we propose a novel packing rule for the multishape part packing problem. The inspiration originates from the old adage “gold corners, silver edges and grass belly” in the Chinese game Go [23, 24], which has accumulated empirically validated rules and wisdom from human experience over thousands of years. This adage indicates that occupying the corner of the board is the most valuable, occupying the edge is second best, and being in the center is the worst; it can apply to the process of part packing as well. In addition, this paper improves the concept of diamond caves and proposes a clever method to quickly find the diamond caves of the parts to be packed.

## The method

In this paper, to illustrate the principle of covering, corner-searching and occupying (C, S&O), three shapes of parts and rectangular bin are used as examples. However, it should be noted that this method is applicable to any shape of parts and raw materials. Next, the principle of this method is introduced in detail in three stages.

### The covering stage of C, S&O

**Fig 6:**
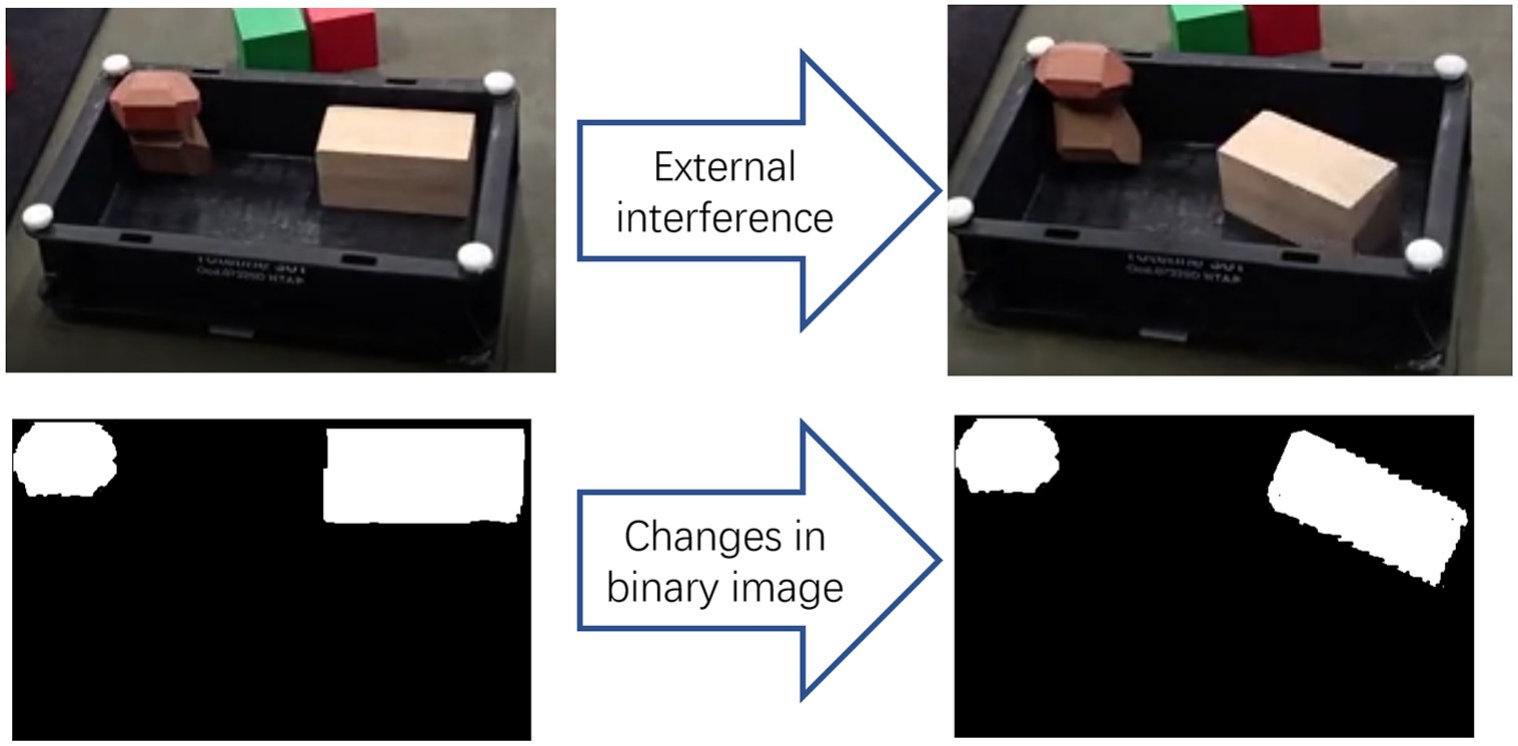
External interference detection. When the distribution of parts in the bin changes due to external interference, the algorithm can still work normally when observed by hand-eye camera.

The main idea of the covering stage is to generate a valid position matrix which indicates all locations in the bin where the sample is captured without overlap. First, the rectangular bin and the part to be packed are transformed into the environment matrix and the sample matrix, where the dimensions of the part are known and the sample matrix is generated directly. In generating the environment matrix, the matrix representation is obtained using the above mentioned image processing method before every packing operation. Using the hand-eye camera to get the top view of the bin, then converting it into a binary image of size *W* × *L* where occupied positions are represented by ‘1’ and unoccupied one by ‘0’. If there is external interference in the working process, such as vibration of the working platform, artificial movement, the traditional algorithm will not continue to work safely. The algorithm proposed in this paper can eliminate the influence with the help of hand-eye camera, making the algorithm more robust. The process is shown in Fig 6. After obtaining the environment matrix and the sample matrix, the sample matrix is convolved with the environment matrix by borrowing the convolutional neural network method of processing pictures, and the valid position matrix ***M***_*vp*_ is obtained.

**Fig 7:**
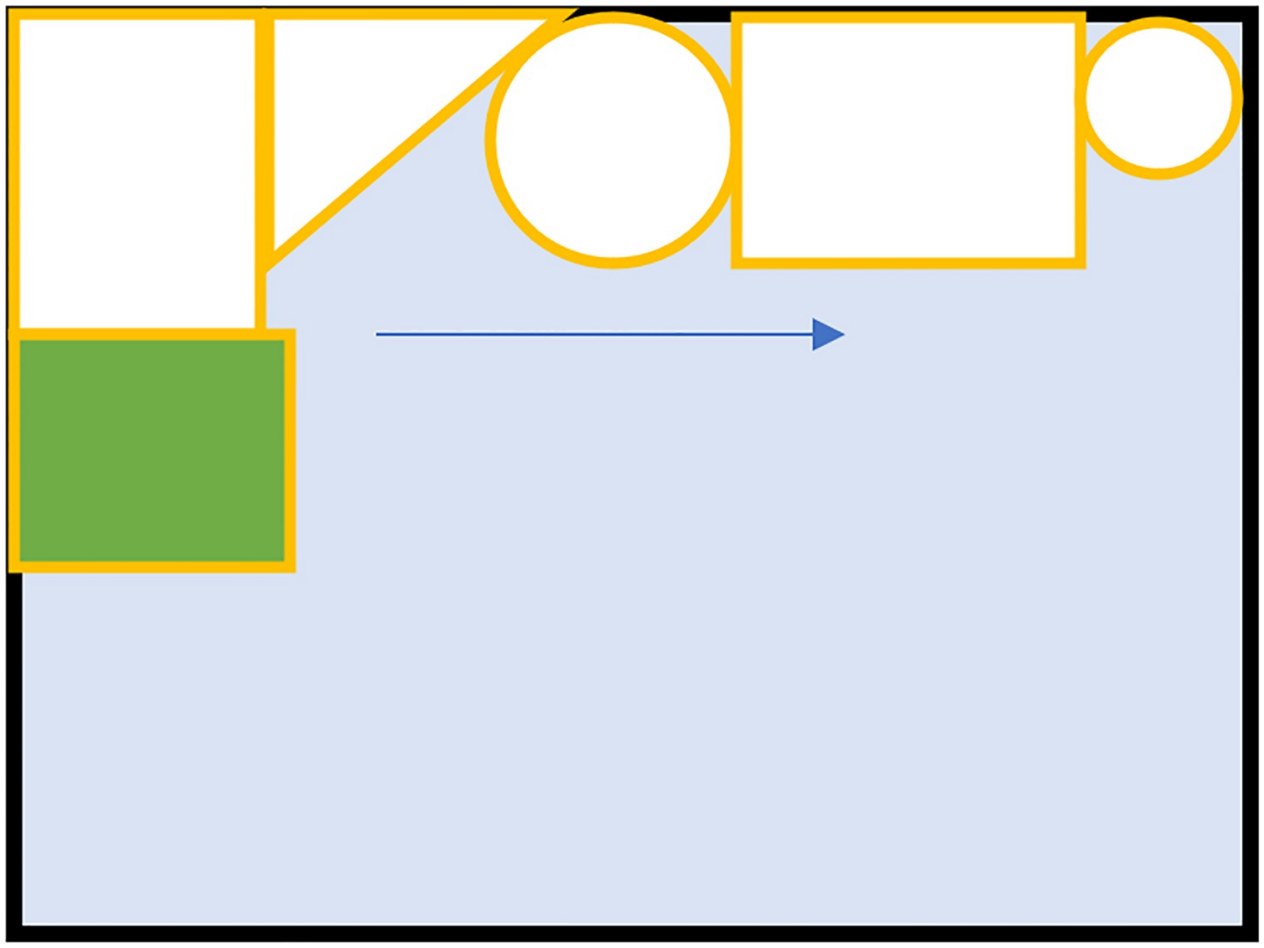
The way to reduce computation. The green part is the part to be packed, and the beige parts are the parts in the bind. To reduce the calculation time, the convolution operation is only performed along the edge of the parts in the bin to obtain the effective position matrix.

Because actual production generally uses the upper end of the rectangular raw material in packing, to reduce unnecessary calculations when seeking the valid position matrix ***M***_*vp*_, the convolution operation is only performed along the upper contour of the packed parts. In the blank portion of the raw material, the valid position matrix ***M***_*vp*_ is automatically completed according to the empirical rule. This method can greatly reduce the time spent in the covering stage. The process is shown in the Fig 7.

### The stage of corner-searching

**Fig 8:**
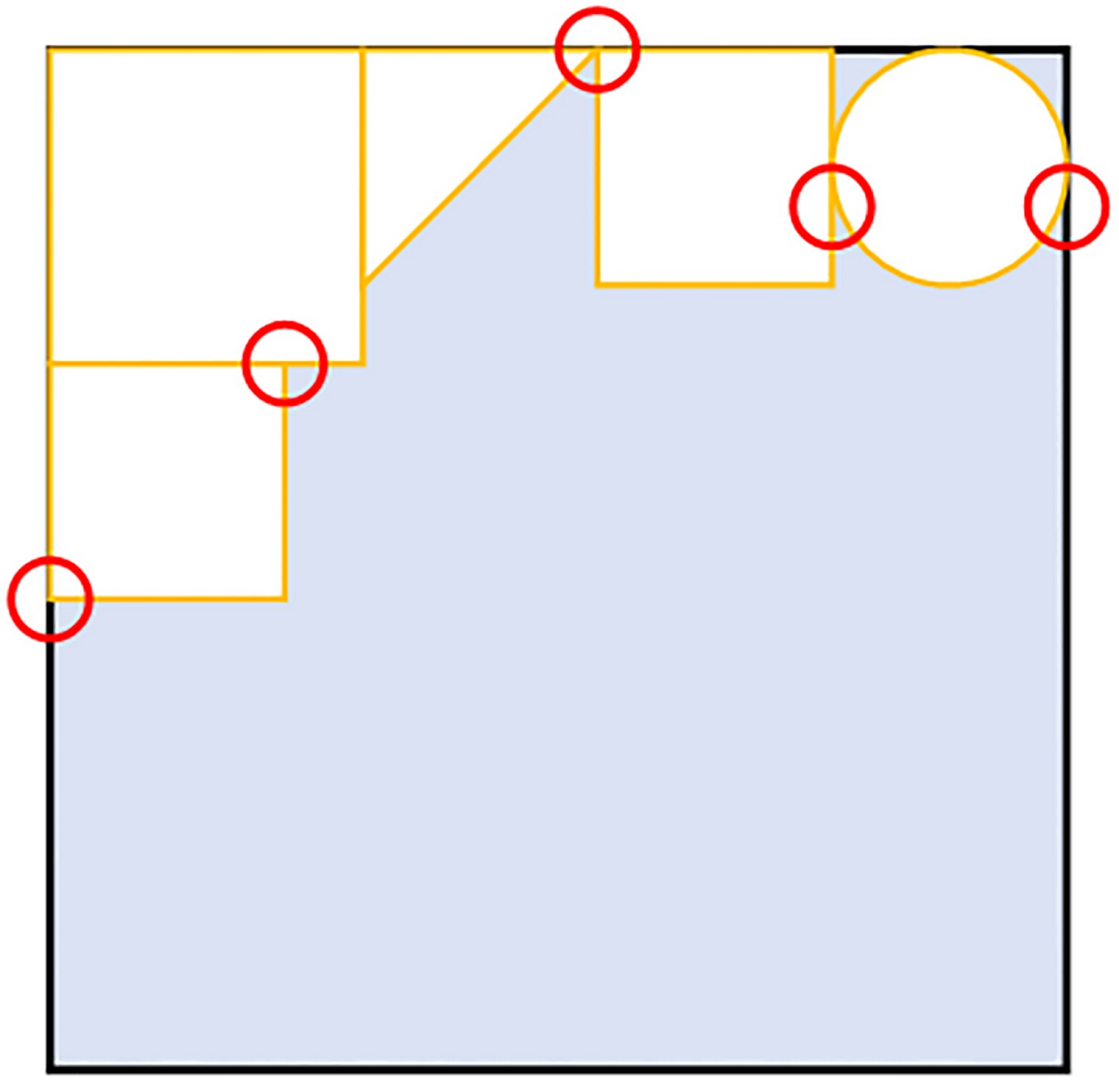
The corner positions. The positions circled in red are the corners in the bin. As the number of the parts in the bin increases, so does the number of corners.

According to the proverb “gold corners, sliver edges, and grass belly”, placing the parts in the corner positions can maximize the use of the raw materials and reduce waste, and at the same time, placing the parts in the corner positions can make the overall pattern neater and speed up the layout process. The definition of the corner position is shown in the following Fig 8

In this stage, the algorithm solves two main problems:

Problem 1: Finding all the corner point locations.
Problem 2: Determining the diamond cave location.

**Fig 9:**
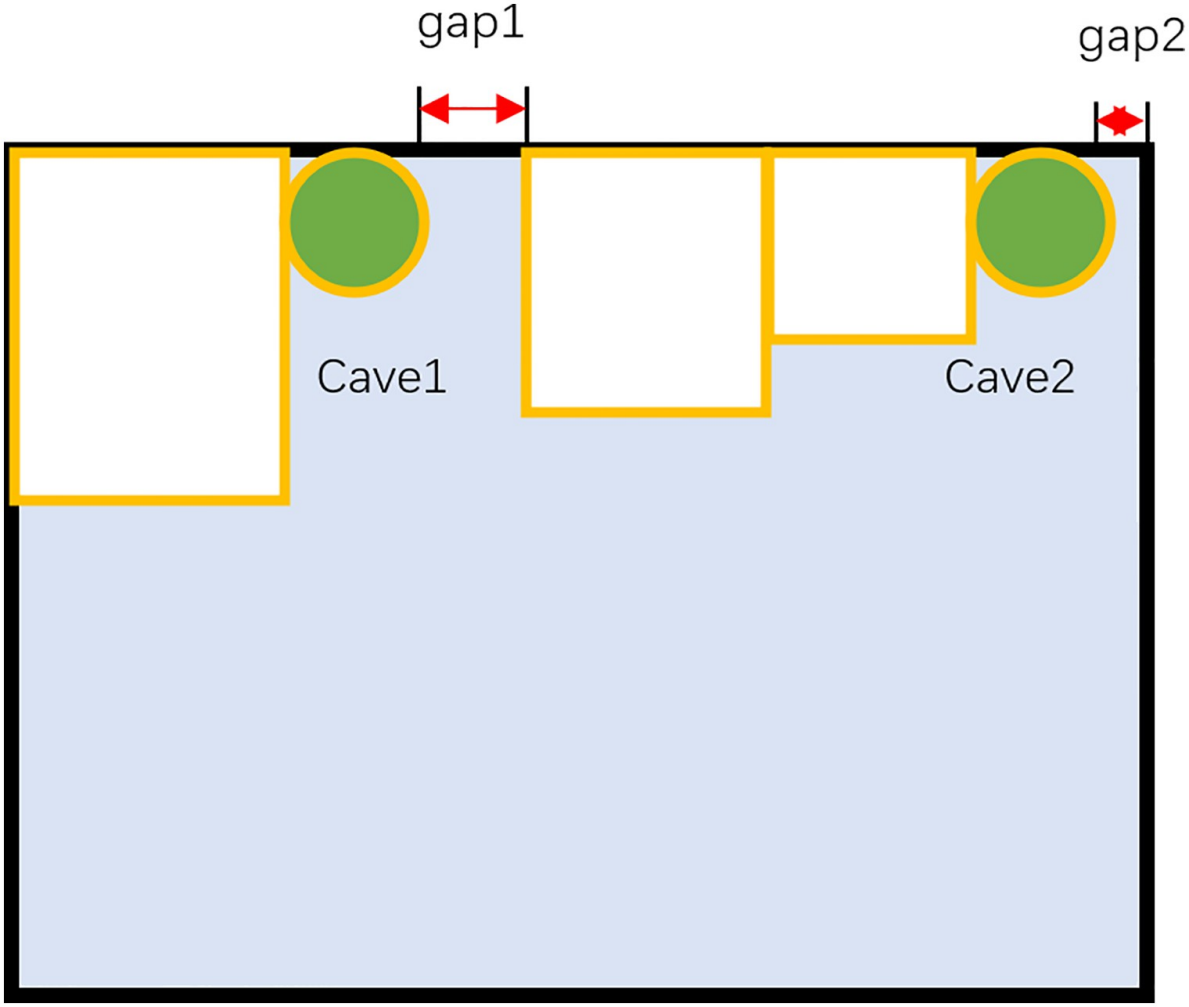
The diamond cave. The white parts are the parts already placed in the bin, and the green circular part is the part to be placed. There are two caves in the bin. Placing the green circular part in cave 2 leaves a smaller gap, so cave 2 is defined as a diamond cave.

It can be noted that for each part layout optimization, there is more than one corner point position on the raw material. And determining the most suitable solution among many corner positions is very significant for the algorithm to approximate the optimal solution. In this paper, the most suitable corner point position is defined as the diamond cave, and the diamond cave refers to the position of the corner point with the smallest gap left after putting in the part. The example is shown in Fig 9. Two caves are formed on the raw material, and the green part is to be packed. Since the gap left in cave 1 is larger than the gap left in cave 2, cave 2 is the diamond cave, and the part should be placed at the corner point in cave 2. The main purpose of this algorithm is to find the location of the diamond cave quickly and accurately before each placement.

**Fig 10:**
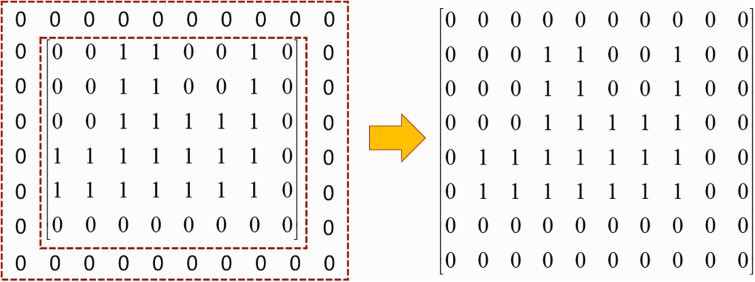
The detection matrix. Padding the periphery of the valid position matrix ***M***_*vp*_ with a circle of 0 elements to obtain the corner point detection matrix ***D***.

Through observation, we find that the valid position matrix ***M***_*vp*_ can be used to get the location of the diamond cave. So this paper proposes a novel method called convolution corner detection(CCD) to detect the current optimal layout position. The steps of the CCD are as follows:

Step 1: The detection convolution kernel is constructed:
  $$\begin{array}{c} C=[\begin{array}{ccc} 1 & 1 & 1\\ 1 & 1 & 1\\ 1 & 1 & 1 \end{array}] \end{array}$$ (3)
Step 2: The periphery of the valid position matrix ***M***_*vp*_ is padded with a circle of 0 elements to obtain the detection matrix ***D***. Taking the valid position matrix in Fig 5 as an example, the padding process is shown in Fig 10
Step 3: ***C*** performs the convolution operation with the detection matrix *D* to obtain the corner matrix *L*, and the position whose element value is less than or equal to 4 in *L* is the corner position. The calculation formula is shown in Eq (4).
  $$\begin{array}{c} L(i,j)=\{\begin{array}{c} \begin{array}{cc} C◦D(i:i+3,j:j+2) & ifM_{vp}(i,j)\neq 0 \end{array}\\ \begin{array}{cc} \text{nvp} & ifM_{vp}(i,j)=0 \end{array} \end{array} \end{array}$$ (4)

**Fig 11:**
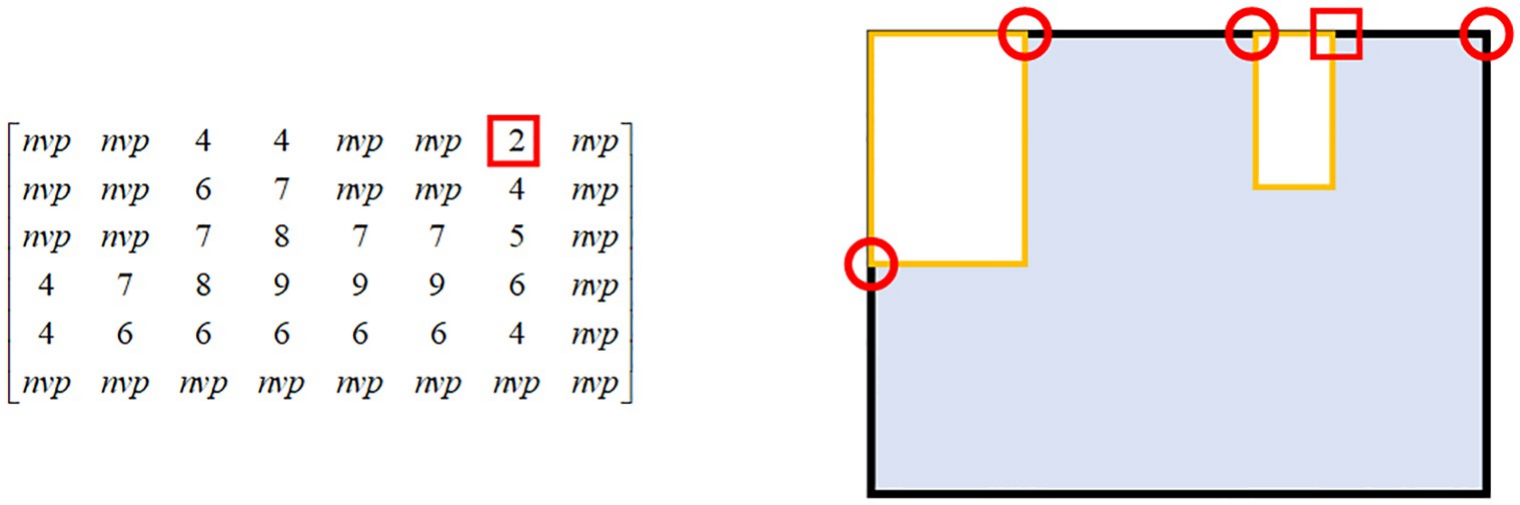
The corner matrix. In the corner matrix, ‘nvp’ means that the position is not valid. The circle indicates the corner position in the current bin, and the square indicates the diamond cave position in the current bin. We can see that the smaller the value of the element, the more suitable the location.

Once the corner point matrix is obtained, all the corner point positions are obtained, as shown in Fig 11. In the corner matrix, ‘nvp’ means that placing part at in that position will occur overlap, and the element with the smallest value corresponds to the location of the diamond cave.

More generally, the size of the coming part varies widely. If the dimension of ***C*** is fixed, there may be many locations with the smallest values, resulting in poor layout performance. To make the algorithm more versatile, we let the size of ***C*** change with the size of the coming part. After several experiments, we tested the effect of ***C*** of different sizes and found that the best generalization is to set the size of the kernel ***C*** to 2/3 of the mean value of the sum of the length and width of the outer envelope rectangle of the part to be packed, and the element values in the kernel are all set to 1. The detection matrix ***D*** is generated according to the size of the part to be packed, too.

At the same time, inspired by the bottom-left heuristic algorithm [12], we aim for the parts to be arranged close to the raw material end, which can not only reduce the difficulty of the layout but also speed up the calculation of the algorithm. After comprehensive consideration, the calculation formula of the corner value is given in Eq (5). The position with the highest corner value is the current optimal layout position selected by the algorithm.

$$\begin{array}{c} V(i,j)=\frac{1}{\sqrt{i^{2}+j^{2}}\times L(i,j)} \end{array}$$ (5)

### Occupying stage

In the first two steps, the diamond cave position of the part to be packed is obtained; however, for rectangular and triangular parts, there may be multiple packing positions for the same part, since there are multiple packing orientations for them. To solve this problem, the new concept of the environment matching degree is proposed to determine correct placement orientation of the parts.

**Fig 12:**
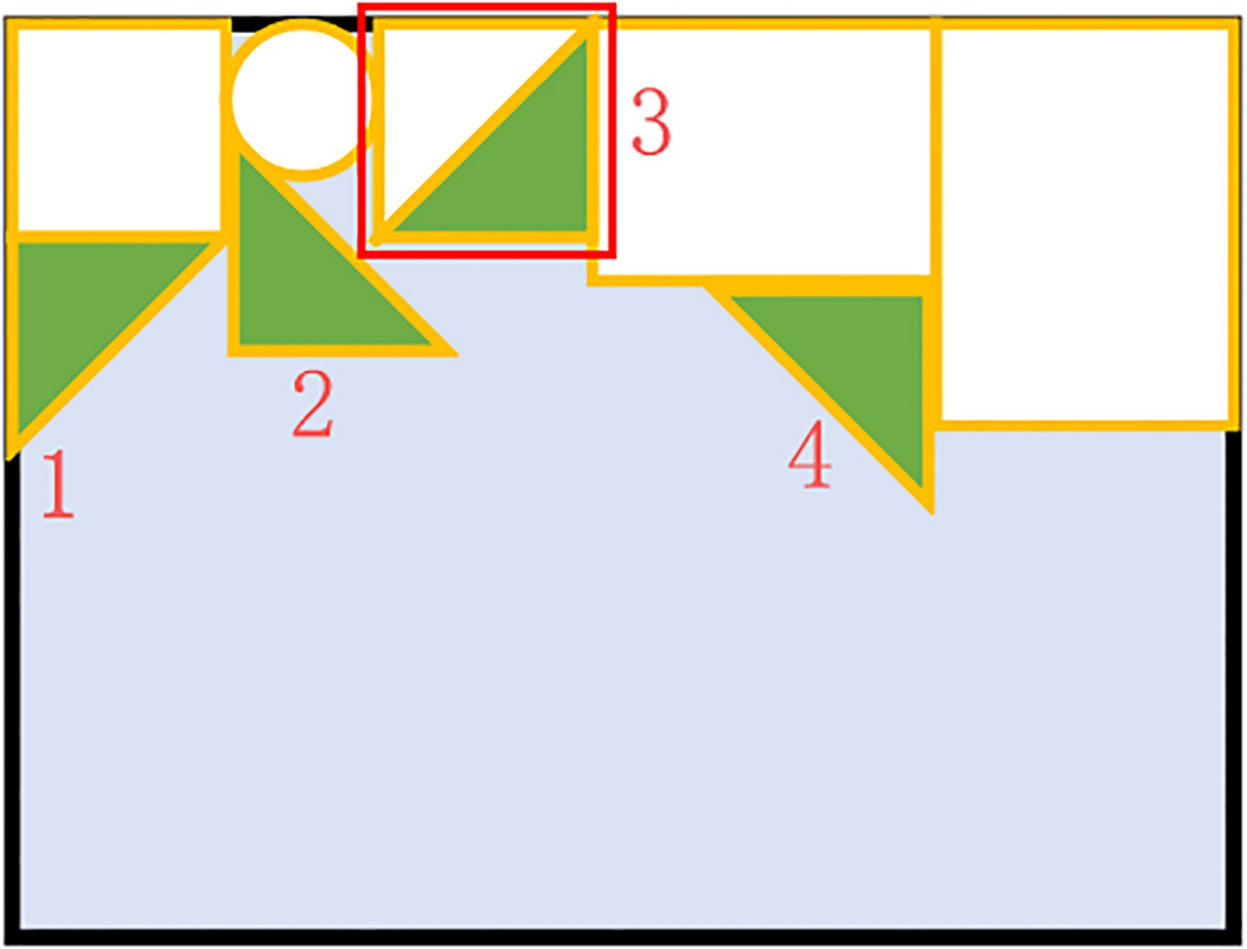
The correct orientation. The white parts are the parts already placed in the bin, the green triangle part is the part to be packed. The triangular part has four layout orientations, and there is a diamond cave in each layout orientation. The most satisfactory placement direction is called the correct direction, The correct placement direction is determined by calculating the environment matching degree.

The environment matching degree is defined as follows: the smaller the cavity area formed between the arranged parts is, the higher the utilization rate of the raw material. Taking the corner value and the cavity area formed by the packing into consideration, the environment matching degree ***Q***_*env*_ is given in Eq (6).

$$\begin{array}{c} Q_{env}=s\times V(i,j)=\frac{s}{\sqrt{i^{2}+j^{2}}\times L(i,j)} \end{array}$$ (6)

where *s* represents the non-cavity area ratio formed by the packing in Eq (6). The calculation method of *s* is shown in the Fig 12. After the positions of diamond caves of the part in all packing orientation is obtained, the fitness *F* for each placement orientation is calculated. Select a rectangular space around the placement position, as shown in the red rectangular box in Fig 12. Calculate the amount of area used in this range and use this value as fitness *F*, the calculation formula of s is given in Eq (7).

$$\begin{array}{c} s=\frac{F-s_{p}}{s_{p}} \end{array}$$ (7)

where *s*_*p*_ is the area of the part to be packed. According to this formula, the advantages and disadvantages of the four layout orientations of the triangular part in Fig 12 can be obtained as: *s*_3_ > *s*_1_ > *s*_4_ > *s*_2_.

The pseudocode is provided in Alg. 1.

**Algorithm 1** Covering, corner-searching and occupying

**Input:** the shape and size (*l*_*i*_, *w*_*i*_) of the part to be packed and the image of the raw material with size (*L*, *W*)

**Output:** pixel coordinates of the current optimal position.

1: Initialize the environment matrix according to the image of the raw material, and initialize the sample matrix according to the size and orientation of the part to be packed.

2: Generate the valid position matrix ***M***_*vp*_

3: Generate the point detection matrix ***D***.

4: **while** *i* < *L* **do**

5:  **while** *j* < *W* **do**

6:   **if** *M*_*vp*_(*i*, *j*) = 1 **then**

7:    ***L***(*i*, *j*) = *M*_*sample*_ ° *D*(*i*:*i*+ *l*, *j*:*j* + *w*)

8:   **else**

9:    ***L***(*i*, *j*) = *nvp*

10:   **end if**

11:  **end while**

12:  then calculate the environment matching degree ***Q***_*env*_

13:  **if** the part has more than one orientation **then**

14:   **Return** to step 1

15:  **end if**

16:  Choose the current optimal placement position with max(***Q***_*env*_)

17: **end while**

18: **Return** the current optimal position

## Experiment

In this section, we present the experimental results to validate the efficiency of C, S&O. First, we set the rectangular raw material size to *W* × *W*. In each iteration during the process, we generate a random part with varying sizes *l*_*i*_×*w*_*i*_ and varying shapes, where $0<l_{i}<\frac{1}{5}W$ and $0<w_{i}<\frac{1}{5}W$, and then we send the part to the algorithm. The algorithm outputs the current optimal position of the part.

Taking into account actual production scenarios, this paper considers two different layout cases: a layout case with complete raw materials and a layout case with damaged and incomplete raw materials.

**Fig 13:**
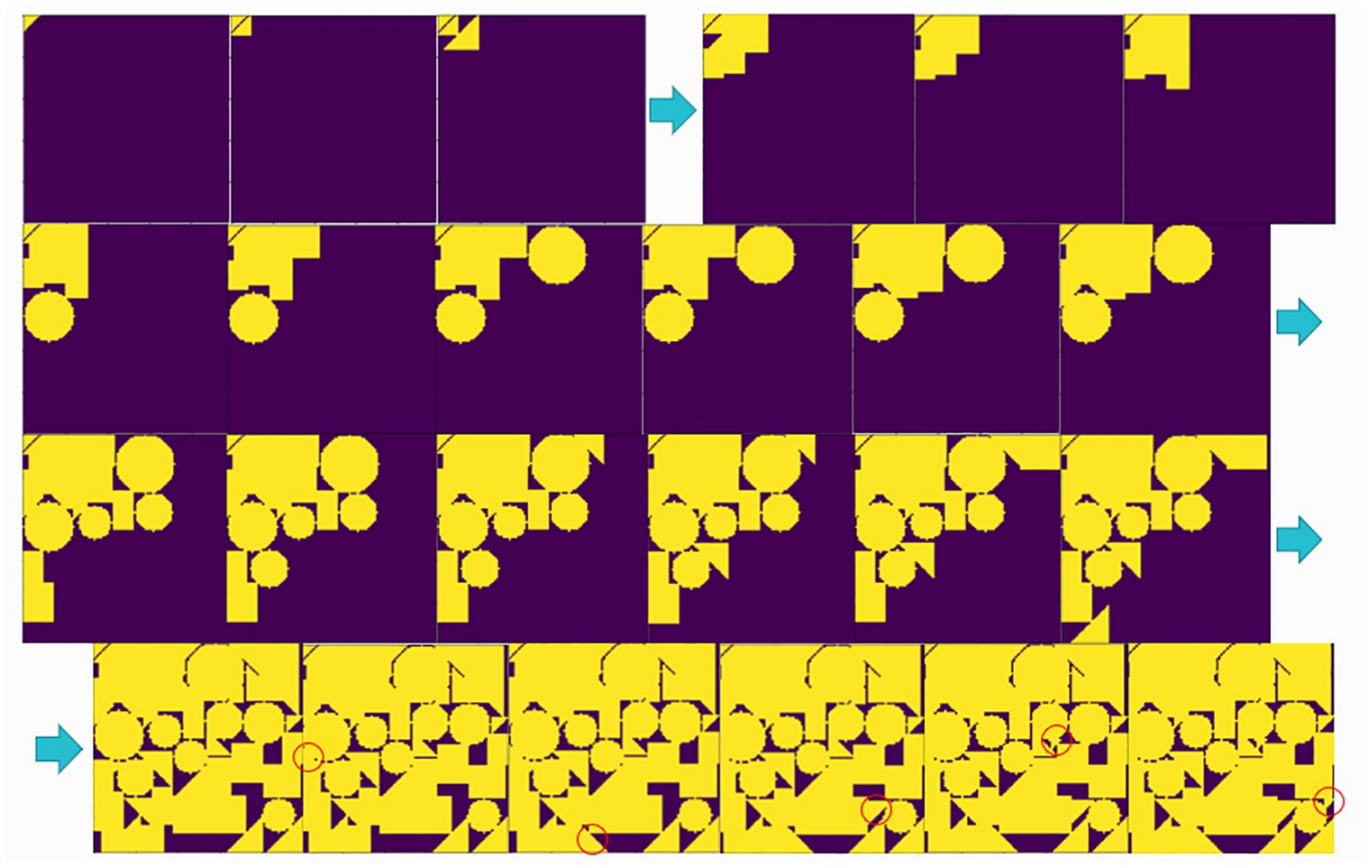
The whole layout process. The whole layout process for complete raw materials.

For the first layout, a complete packing process case is shown in Fig 13, which mainly shows the steps that can highlight the intelligence of the algorithm and omits some simple packing steps. The simulation experiment was conducted on a self-contained laptop with an AMD R7 4800 processor running with 16 GB operating memory. In the simulation experiment shown in Fig 13, a total of 55 parts were randomly generated, with a total layout time of 25.7118 s and a raw material utilization rate of 86.68%, and the average payout time of each part was 0.4675 s.

After many experiments, the average utilization rate of the raw materials in the first layout case is 85%, the layout time is 25 s, and each piece of raw material can have approximately 50 parts arranged on it.

**Fig 14:**
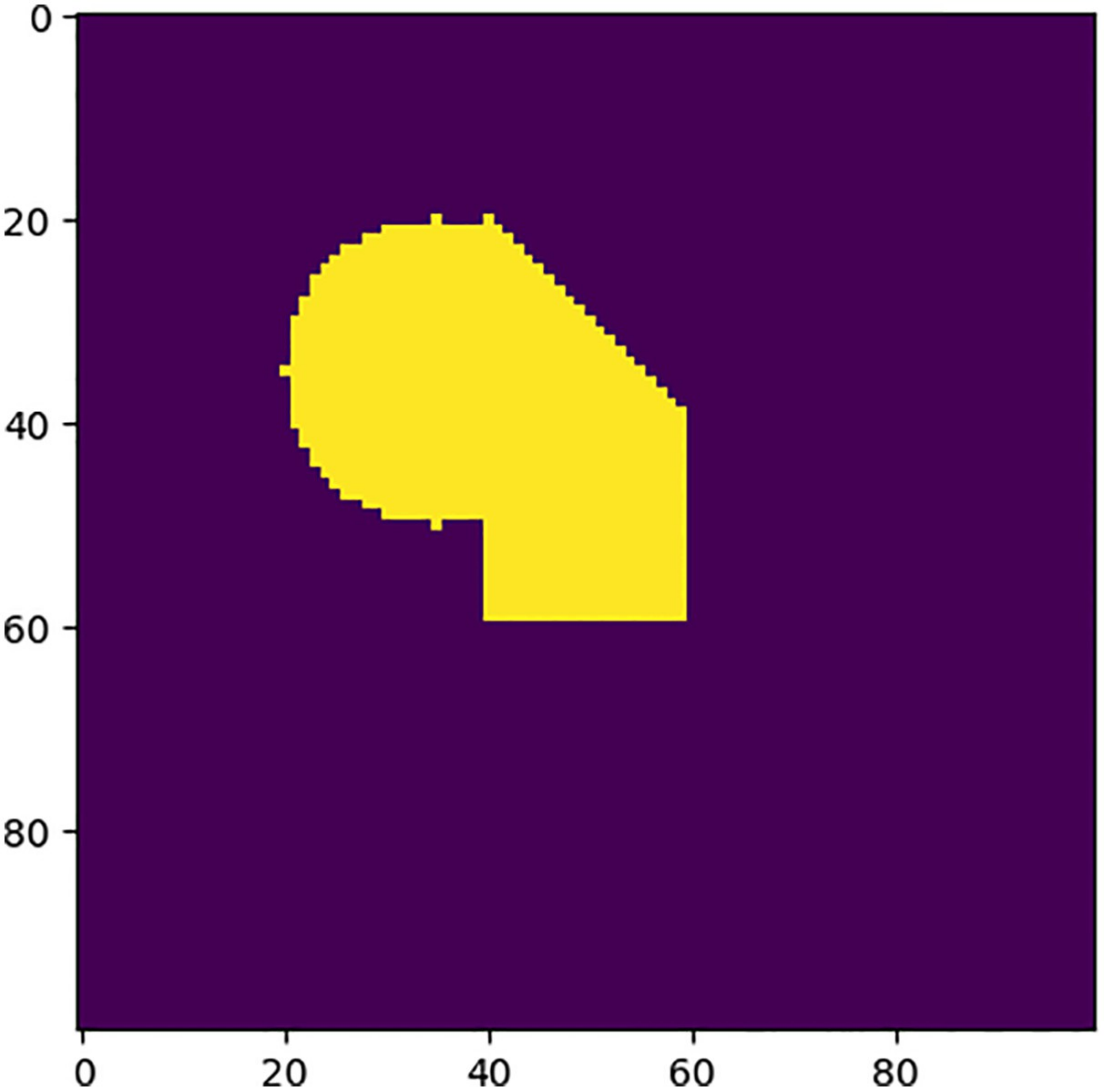
The non-empty bin. In the figure, the dark area is the part of the material that can be used, and the light part of the raw material is destroyed.

In the second layout case, we randomly generate damaged and incomplete raw material for layout optimization, as shown in Fig 14. This algorithm can process damaged raw material of any shape with the help of image processing technology, which provides the possibility for industrial production to eliminate manual layout completely.

**Fig 15:**
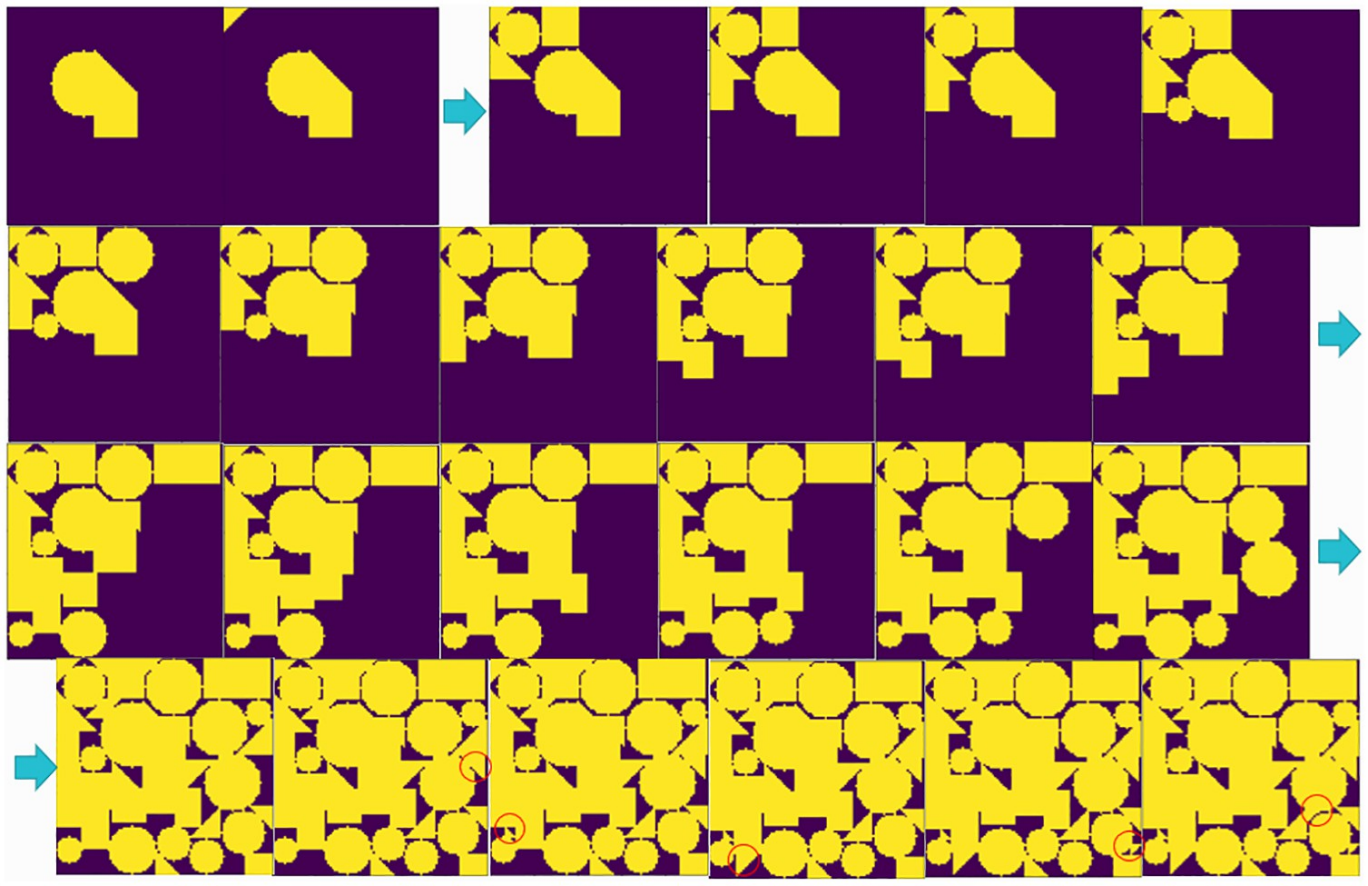
The whole layout process in the non-empty bin. The whole layout process for damaged raw materials.

The layout process given by the algorithm is shown in Fig 15. A total of 50 parts were laid out, which took 20.783 s, and 83.1385% of the raw material was utilized. The average time of each part was also less than 0.5 s. Through the above cases, it can be determined that the algorithm can process raw materials of any shape with the help of image processing technology.

**Fig 16:**
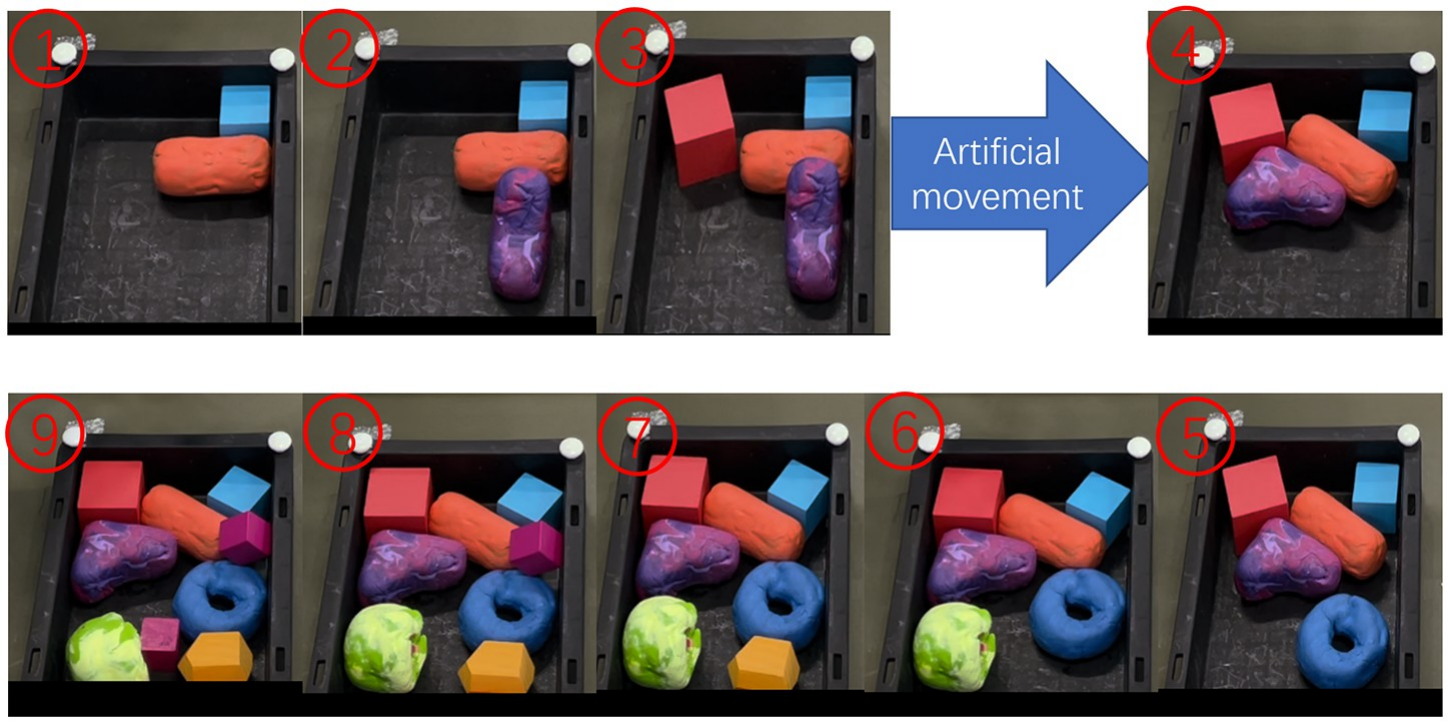
The whole process of the physical experiment. The agent obtains the layout information of the bin through the hand-eye camera. During the assembly process, the original layout of the bin is artificially disturbed, and the agent still works normally.

In order to verify the effectiveness of the algorithm, a physical simulation is carried out. In this experiment, the mechanical arm is UR 5e robotic arm, the hand-eye camera is Realsense D435, the result is as shown in Fig 16. You can see that the algorithm works even if there is artificial movement of the parts in the bin. The average time of each part is also less than 0.5 s.

## Conclusion

In this study, we present a new three-stage methodology, called covering, corner-searching and occupying, to obtain satisfactory solutions for the two-dimensional multishape part packing problem. To explain the principle of the algorithm more clearly, this paper takes ore collection task as an example. The algorithm can complete this task for parts of any shape with the help of image processing technology. The C, S&O method uses a matrix representation of the parts and raw materials, cleverly uses convolution operations for overlap detection, and uses a dynamic corner point detection convolution kernel to search for the location of the “diamond cave” by drawing on the corner occupancy principle, taking into account that parts of different shapes have different packing orientations and defining the new concept of the environment matching degree. By finding the corner point with the maximum environment matching degree, we determine the satisfactory placement position and orientation of the part to be packed.

In this paper, several sets of experimental simulations are conducted, and the experimental results verify that the algorithm is able to quickly deal with the problem of packing multishape parts. Additionally, owing to the preprocessing of raw materials by image processing technology, the algorithm is able to plan the layout of arbitrarily shaped raw materials. The next research will focus on extending the ideas of this algorithm to the 3D bin packing problem (BPP).

## Supporting information

S1 Video The whole layout process for complete raw materials. (MP4)

S2 Video The whole layout process for damaged raw materials. (MP4)
